# Acceptability of using the medication monitor and experience of a differentiated care approach for TB treatment adherence among people living with TB in South Africa

**DOI:** 10.1371/journal.pgph.0001885

**Published:** 2023-10-27

**Authors:** Rachel Mukora, Barack Ahumah, Noriah Maraba, Catherine Orrell, Lauren Jennings, Pren Naidoo, Katherine L. Fielding, Kavindhran Velen, Salome Charalambous, Candice M. Chetty-Makkan

**Affiliations:** 1 The Aurum Institute, Aurum House, Parktown, Johannesburg, South Africa; 2 University of Witwatersrand, School of Public Health, Johannesburg, South Africa; 3 Department of Medicine, Institute of Infectious Disease and Molecular Medicine, University of Cape Town, Cape Town, South Africa; 4 Desmond Tutu Health Foundation, Cape Town, South Africa; 5 Stellenbosch University, Stellenbosch, South Africa; 6 London School of Hygiene & Tropical Medicine, London, United Kingdom; 7 Faculty of Health Sciences, Health Economics and Epidemiology Research Office, University of the Witwatersrand, Johannesburg, South Africa; ICMR-National Institute of Epidemiology (Chennai, India), Department of Health Research, Ministry of Health and Family Welfare, Government of India, INDIA

## Abstract

**Background:**

The introduction of digital adherence technologies (DATs) such as medication monitors in tuberculosis (TB) programmes supports treatment adherence among people with tuberculosis (PWTB). We evaluated the acceptability of using medication monitors (Wisepill evriMED) prompting a stepwise differentiated care approach (DCA), involving short message service (SMS), phone calls, home visits and motivational counselling, among PWTB in South Africa.

**Methods:**

We conducted 62 in-depth interviews with participants in local languages across three provinces (January—October 2020), purposively selected by treatment month, adherence history and gender. Interviews were audio recorded, transcribed verbatim and translated. Using a deductive approach and the Theoretical Framework for Acceptability (TFA), we explored acceptability across the sample attributes.

**Results:**

PWTB across adherence histories showed a positive attitude to using the evriMED device and receiving the DCA support. PWTB described the SMS reminders and phone calls as effective reminders, though home visits were less acceptable, due to perceived stigma. Despite willingness to participate in the intervention, the large size of the monitor and sound of the alarm drew attention, potentially causing embarrassment and stigma. Due to perceived stigma, some PWTB adapted the intervention by leaving the monitor at home after removing the pills to ensure that someone else tracked usage, while the PWTB used alternative reminders such as cell phones to take their medication.

**Conclusion:**

Although PWTB showed a positive attitude towards the intervention, perceived stigma contributed to participants adapting their lifestyle to meet treatment adherence requirements without using the monitor. However, the medication monitor was a tool that seemed to prompt this personal change in behaviour. Achieving people-centered TB care, including the introduction of DATs, will require that TB programmes incorporate PWTB insights to maximize their use and effectiveness.

## Introduction

The treatment success rate for drug sensitive Tuberculosis (DS-TB) in South Africa remains concerning at 78% for new care recipients and people with TB (PWTB) that relapse [1],whereas the global target is set at 90% [2]. Reduced treatment success rates are due to sub-optimal treatment adherence, catalysed by stigma, forgetfulness, lack of support from friends and family, distance from clinics, lack of counselling and poor health worker-patient relationships [3].

The introduction of digital adherence technologies (DATs), such as medication monitors in TB programmes may support treatment adherence among PWTB. Medication monitors that remind PWTB to take their medication are less resource intensive and can monitor medication dosing patterns in real time [4]. Differentiated care approach (DCA) is a person-centered approach where services are adapted to the needs of PWTB, helping to shift utilization of resources to those who are in most need [5–7]. Medication monitors allow for efficient use of health system resources where allocation of resources can be re-directed to those who miss doses by offering tailored care packages in the form of differentiated care services [8].

Nevertheless, some medication monitors are bulky and have a loud alarm which may lead to unintentional disclosure of TB status thus interfering with adherence [9]. Despite this, one pilot study in KwaZulu-Natal (KZN) province SA, showed that use of medication monitors did not cause stigma or compromise confidentiality when pills were concealed [10]. The KZN study took place over three weeks period with hospitalised drug-resistant TB (DR-TB) individuals. Little is known if the findings from that study [10] would be similar amongst out-patients using medication monitors over a longer duration. Although, there is limited information on effectiveness of using a DCA, PWTB perceived healthcare workers (HCWs) as supportive when patient medication doses were monitored remotely [10].

Adoption of medication monitors relies on understanding end-user insights to refine implementation. However, there is limited evidence on the acceptability of medication monitors when combined with a DCA in PWTB. A formative study from Uganda where 35 PWTB were interviewed to elicit perceptions of using electronic monitors feelings about monitoring medication intake using a medication monitor (Wisepill device) and receiving SMS reminders supported acceptability. Real-time adherence interventions could potentially provide acceptable approaches to remind and motivate PWTB to take medication regularly. However, findings were limited since participants were asked about their perceptions before they could use the intervention in real life [11]. The care package in this study was limited to only SMS reminders.

As little is known about the lived experiences of end-users of DATs and DCA in PWTB, we evaluated the acceptability of using medication monitors (Wisepill evriMED 1000 device) prompting a DCA. The DCA involved short message service (SMS), phone calls, home visits and motivational counselling, among PWTB in South Africa. This evaluation was conducted as part of the TB Monitoring Adherence to Treatment Endpoints (TB MATE) study.

## Methods

### Study design and study setting

This exploratory qualitative study was nested within a cluster-randomized trial (CRT) implemented in 18 clinics across three provinces of South Africa: Gauteng (Ekurhuleni district); Western Cape (Klipfontein and Mitchell’s Plain districts); and Kwa-Zulu Natal (eThekwini district) [8]. In the intervention arm of the CRT, participants received medication monitors with daily timed alarms and lights serving as reminders to take their medication incorporating a DCA in response to missed doses identified from a central database [8]. The alarm time was configured at enrolment as per the participants preference, but the alarm’s volume and light intensity could not be adjusted. The DCA was implemented in a progressive manner depending on the number of doses a participant missed [8]. If one dose was missed, then a SMS reminder was sent to the participant [8]. If a second or third dose was missed, then study staff contacted participants telephonically; a home visit was conducted when the fourth dose was missed to provide motivational counselling [8]. An additional light on the medication monitor served as a reminder for monthly dispensing visits [8].

### Site selection

The qualitative study took place in the nine intervention clinics, three in each province. Clinics were selected based on location, HIV prevalence and total number of PWTB starting TB treatment per month [8]. Adult HIV prevalence in the general population is 12.5% in Gauteng, 8.9% in Western Cape and 18.2% in Kwa-Zulu Natal province, according to the 2017 National HIV Prevalence, Incidence, Behaviour and Communication Survey [8,12].

### Study population

The study population included men and women that were enrolled in the CRT, had drug sensitive TB disease and who are initiated on TB treatment, aged 18 years and older, and fell into adherence categories (green, orange or red). Those in the green category received an SMS, orange category received a phone call while those in the red category had received a home visit (Table 1). Participants were purposively selected by treatment month, gender and weekly adherence history for equal distribution across these attributes to ensure variation in the responses.

**Table 1:** Interventions.

Weekly Adherence	Weekly Doses missed	Adherence category	Intervention
85%	1	Green	Same day SMS reminder (automated)
45–84%	2 or 3	Orange	Phone call by nurse or counsellor
<45%	4 or more	Red	Home visit and/or clinic visit
Greater than one week with 4 or more missed doses			Recall for other adherence measures e.g. motivational counselling.

### Conceptual framework and themes explored

**Fig 1:**
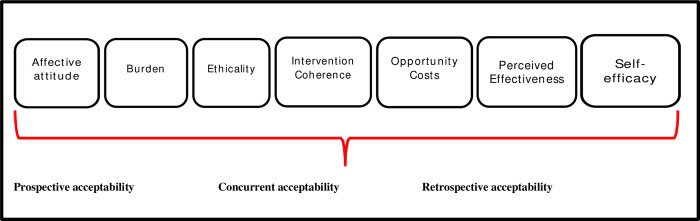
Theoretical Framework of acceptability (Sekhon et al 2017 [13]).

We used the Technology Acceptance Model (TAM) [9] to develop an in-depth interview (IDI) guide that explored the following themes: (i) level of satisfaction with activities related to the differentiated model of care and use of the medication monitor technology; (ii) feelings and attitude toward use of the technology; (iii) reasons for accidental opening of the monitor (iv) ease of the use of the monitor; (v) perception and experience of receiving SMS’s, home visits and motivational counselling; (vi) stigma; (vii) cultural or other barriers to uptake of the differentiated model of care. Using the Theoretical Framework for Acceptability (TFA) [13] shown in Fig 1 below, we explored prospective, concurrent, and retrospective acceptability (before, during, and after the intervention) across the sample attributes. We used two models as the emergence of inductive codes meant that the TAM was not adequate, and these codes would be best summarised using the TFA.

### Data collection

From January to February 2020, we piloted the IDI guide with six participants, two males from the green category, two females from the orange category and one female and one male from the red category. Interviews were conducted face-to-face at a private area within the clinic and the data was included in the analysis. Minor adjustments were made to the initial guide to include additional background demographic questions. Data collection continued between July and October 2020. Each interview took 45–90 minutes. All interviews were conducted in the preferred local language of the participant by a male or female research assistant with qualitative training and experience who was accompanied by a note taker (either male or female). The researchers did not establish a prior relationship with the participant, though they carefully explained the information sheet enabling them to understand the reasons for the study. The interviews were digitally recorded following consent and transcribed verbatim by trained research assistants. The transcripts were not returned to the participant for comment or correction. Only the participant, interviewer and note taker were present during the interviews. Limited authorised staff and study investigators had access to information that could identify individual participants during or after data collection. Saturation was assessed during data collection through debrief sessions with data collectors, by asking the same question in different ways and reviewing a sample of the recordings until no new information emerged. No repeat interviews were carried out.

### Data analysis

We used thematic analysis including deductive and inductive approaches [14,15]. At least 10% of the transcripts were coded by two independent researchers (BA and RM) to reduce bias and improve reliability [14]. A codebook of emerging themes was developed, guided by the framework on the ‘Theoretical Framework for Acceptability’ [13]. Where there was no inter-rater agreement, the theme was dropped [14]. The final codebook was used to code the remaining transcripts and any new codes that emerged were included. MAXQDA qualitative software was used for the coding process. We present the major themes that emerged and use supportive direct quotations from participants. Participants did not provide feedback on the findings.

### Ethics approval and participant consent

The parent study received ethics approval from the three district and provincial ethics committees where the trial sites were located. This qualitative study was approved by the Human Research Ethics Committee (HREC) of the University of Witwatersrand (Ref 180705) and the University of Cape Town (Ref 452/2018). We obtained written informed consent for study participation and permission for digital recording from all participants. Participants were reimbursed for their time and transport related to the interview.

## Results

We selected 85 PWTB, and 62 were interviewed. Twenty-three 23 PWTB either declined to participate (n = 6), could not be reached (n = 12) or were not fit to participate (n = 5). Out of the 62 interviews conducted, we analysed 61 in total across 3 provinces. One interview was excluded from the analysis since the participant was below 18 years and did not meet the inclusion criteria. Across the three provinces, majority (55.74%) of the PWTB were male (n = 34). Table 2 below shows the distribution of the treatment month and the adherence categories.

**Table 2:** Characteristics of study participants.

	Gauteng (n = 20)	KwaZulu-Natal (n = 21)	Western Cape (n = 20)
Female	9 (45%)	6 (29%)	12 (60%)
Treatment month <2months	2 (10%)	4 (19%)	5 (25%)
Treatment month <2–6 months	18 (90%)	17 (81%)	15 (75%)
Adherence category • Missed 1 dose a week (Green) • Missed 2 or 3 doses a week (Orange) • Missed 4 or more doses a week (Red)	5 (25%) 8 (40%) 7 (35%)	13 (62%) 6 (29%) 2 (10%)	7 (35%) 7 (35%) 6 (30%)

Major themes that emerged on the use of the monitor and the DCA that aligned to the TFS framework constructs [12] included: (1) positive attitude towards using the medication monitor and receiving the DCA support; (2) burden and adaptability to the medication monitor; (3) intervention coherence and self-efficacy; and (4) perceived effectiveness of the intervention.

### Major Theme 1: Positive attitude towards using the medication monitor and receiving the DCA support

PWTB across adherence categories showed a positive attitude towards using the monitor and receiving the DCA support. The quotations below show that phone calls and SMSs were well accepted as PWTB felt that providers cared for their health.

*“There is nothing I don’t like about the box. I like this box a lot because it helped me a lot, if it wasn’t for it, I don’t think I would be here today.”–**Female, 23 years, Gauteng, Orange category***

*“I liked being called and being sent SMSs and it might happen that you have forgotten to take your pills and you’d be reminded by being called and asked why you didn’t open the box*… *Then you will go take your pills and remember that you had not opened the box*.”*—****Female*, *37 years*, *KwaZulu-Natal*, *Orange category***

*“It felt good knowing that there were people who cared for me and were looking out for my health besides my family*.”—***Male*, *36 years*, *Western Cape*, *Red category***

However, few PWTB were upset due to the timing of the text message and home visits especially among those who perceived stigma by their neighbours.

*“I don’t want to lie. I didn’t like those SMSs. They irritated me because I had taken them at 9. You’ve opened it [referring to the medication monitor] and took pills, then closed it.”–**Female, 25 years, Western Cape, Green category***

*“It [referring to home visits] made me to be feel shy. It made me think as if they are gossiping about me or all things of sort that was what I was thinking.”–**Male, 18 years, KwaZulu-Natal, Orange category***

### Major Theme 2: Burden and adaptability to the medication monitor

Some PWTB did not like the monitor alarm and made alternate arrangements to take their medication as described in the quotations below.

*“In the first week I was using this box… when I am on the way or I am in a taxi and it rings at 09:00, I would be embarrassed. I would open and close it. The person sitting next to me would be alarmed by what is ringing in this persons’ bag.”–**Male, 37 years, Western Cape, Red category***

*“It is just that it once happened that… it went off in their [referring to friends] presence, they did not have a problem with the noise, it was not that annoying, it’s just that I did not want them to know that I take treatment for TB.”**–Male, 30 years, KwaZulu-Natal, Green category***

*“I will not take it wherever I go*. *When I am visiting a relative to sleep there and come back tomorrow*, *I leave the box at home*. *I will not go around with the box*. *The one who is at home I tell him that when the box ring at 09*:*00 open it and close*. *They do what I tell them to*, *it is like I am with them*. *When I open it and close it*, *it has recorded that I have taken my medication*.*”*–***Male*, *67 years*, *KwaZulu-Natal*, *Green category***

There were mixed views on features of the medication monitor described by the quotations below.

*“So the only thing I don’t like about it is that it’s not very mobile neh so it’s like if I have like somewhere to go to…and I don’t need anything else besides my phone, my phone is this size, the bag I’m carrying is literally that size as well… I don’t have enough space so the only problem I have with it is, I can’t carry on with it everywhere I go, it’s big.”—**Female, 21 years, KwaZulu-Natal, Orange category***

However, some PWTB had no concerns with the loud alarm and large size of the monitor

*“No, I am not bothered as to what people have to say because it is for my health and they will always talk and that is all they can do, talk.”–**Male, 25 years, Western Cape, Orange category***

*“This is my life*, *I can’t hide this box or hide my medication because I’m around people at work*, *visiting relatives or anywhere*.*”*–***Male*, *31 years*, *Gauteng*, *Red category***

### Major Theme 3: Intervention coherence and self-efficacy

Most participants understood the intervention, how it worked, and were able to describe the meaning of the lights fitted on the monitor. The participants described the monitor as a—"smart box", “alert box”, "lunch box", "amazing", "container” (because of its large size), “piano" or “big phone” (because it rings).

*“I also call it lunch box. I say “give me my lunch box, I want to take my medication.”*

*–**Female, 33 years, Gauteng, Red category***

*“This box really helps because when it is time to take your medication, it reminds you and you also decide that you are too scared that it might alert the clinic that you did not open the box or whatever. It was one of the things that I was personally afraid [mindful] of.”–**Male, 37 years, Western Cape, Red category***

*“You just tell yourself: “you know what? Let me just drink my medication and… I will… live a healthier life. So that’s what I want”.–**Male, 37 years, Gauteng, Green category***

Even though some participants felt like they were being watched, the monitor helped these participants adhere to treatment.

*“This box helps me. I don’t go out without taking my medication. This box must ring first before I go out. It must ring first before I go out.”**–Male, 49 years, Gauteng, Orange category***

*“I never felt [found] ashamed or my TB situation whatsoever because I also get support I also know that…as long as I follow instructions from the clinic about medication so they always support me by calling me that’s the support I get from the clinic.”*

*–Male, 43 years, Gauteng, Orange category*

The participants understood that if they did not take their medication, then an SMS, phone call and home visit would follow.

*“What I see, if I forget to take the pills, at the clinic they call, they send me an SMS reminding me. If they see that I haven’t taken them the next day they call.”**–Female, 37 years, KwaZulu-Natal, Green category***

*“No, I was told that if I do not take my medication once or twice that they will send me a message. If I do not take it after that then they will give me a call or come to my house.”–**Male, 25 years, Western Cape, Orange category***

The PWTB felt confident that they could perform the behaviors required to participate in the intervention and they showed willingness to participate due to the perceived benefits.

*“I was then asked if I would not have any issues if I used this box. I said, no I would not have a problem because it would help me in the long run.”**–Male, 37 years, Western Cape, Red category***

### Major Theme 4: Perceived effectiveness of the intervention

PWTB accepted the intervention due to their desire to get healed and not spread TB to close contacts, as supported by the quotations below.

*“Eeh because I wanted to get healed and I didn’t want TB to have more powers and even people I stayed with get infected.”–**Female, 48 years, Gauteng, Orange category***

*“At first, I was quite surprised. I wondered why they would give me a box instead of just the treatment, but after being told about its purpose, I realized it would be helpful and my family was supportive about it at home.”**–Male, 36 years, Western Cape, Red category***

*“…I can feel that I am better than before, I was sick but now I can feel that I am getting better I can walk which means the box is working.”**–Male, 36 years, KwaZulu-Natal, Green category***

It was apparent that PWTB placed high value on the monitor, with some using it as a safe storage not only for their TB medication but also for other personal valuable items.

*“I am imp- impressed for having the box in such a way that, I even place my I.D [referring to identification document] inside and my other valuable items everything.”**–Female, 19 years, KwaZulu-Natal, Green category***

Even those who were in the red adherence category and had missed a few doses still appreciated the technology and the additional reminders from the SMS notifications.

*“I don’t know how it notify them but I feel that they are able to find out in some way. If my box didn’t ring they are able to see that I’m not taking my medication correctly, this box is a proof that am I doing the right thing by taking my medication.”**–Male, 31 years, Gauteng, Red category***

*“But the SMS…because as I might add that I am always with my phone. When it says “ti-ti” it just means something, I say “oh” so fast and immediately I remember.”*

*–Male, 30 years, KwaZulu-Natal, Green category*

Home visits were viewed by some PWTB as being beneficial not only to themselves but also to the entire family. The desire to share the benefits of the intervention was also seen through PWTB who recommended friends and family with symptoms of TB to ask for the monitor at the clinic, if they received a TB diagnosis.

*“I don’t mind them [HCWs] coming home because it’s not just me who benefit, my family benefits as well.”–**Female, 37 years, KwaZulu-Natal, Green category***

*“All I can tell him/her is that they must go to the clinic and find out if he/she has TB and ask for the box. This box will help him/her remember his/her dates and will also help her keep his/her pills safe, undamaged and not lost.”**–Female, 37 years, KwaZulu-Natal, Green category***

## Discussion

Our study found that the use of medication monitors (Wisepill evriMED 1000 device) with a differentiated care approach (DCA), was acceptable among PWTB. Through the four major themes which emerged from our study, we found that PWTB had a positive attitude to both the monitor and the DCA. They appreciated the reminders, social support and assistance with accepting their TB diagnosis. This finding is similar to the study from Kwa Zulu-Natal where participants reported positive experiences from using the electronic pillbox [10]. Likewise, the formative study done in Uganda found that the medication monitor, an earlier version of Wisepill device, was acceptable and participants felt that being monitored enabled them to demonstrate their commitment to adherence [11].

However, we found that some people felt burdened by the idea of moving around with the monitor due to the alarm sound and size which could lead to disclosure of their TB status. A similar finding was seen in India amongst Multidrug-resistant TB (MDR-TB) patients using a similar medication monitor (the Medication Event Reminder Monitor—MERM) where lower acceptance of the medication monitor was related to its large size causing difficulties in portability and storage [16]. The PWTB in India also removed the medication from their device to avoid any stigma and discrimination when moving around [16]; and recommendations suggested that future improvements to the design of the medication monitor could improve the consistent use of the medication monitor [16]. Optimizing the design of the medication monitor is a recommendation that has also been cited as a means to improving acceptability in earlier versions of the MERM that were used to improve adherence to antiretroviral therapy (ART) [17]. Various design options related to the monitor’s size and alarm should be considered based on the preferences of different groups of PWTB such as those who are homebound, travellers, stigmatized etc.

We found that PWTB in our study had good intervention coherence and self-efficacy as they understood how the monitor worked and what to expect if they did not open it upon the alarm ringing. Most of the participants found the monitor easy to use and felt that they should “obey the rules’ not only out of fear that the provider was watching them but also because they were keen to live a healthier life. They felt confident to perform the required actions and found the monitor and DCA effective in helping them adhere to treatment. This finding is different from the study done in India where PWTB had incorrect understanding of the MERM due to sub-optimal counselling which may have led to incorrect use of the medication monitor [16]. However, a recent meta-analysis of a survey conducted in six countries showed that PWTB had a favourable impression of their capability to use DAT (including Wisepill evriMED in Ukraine and South Africa) which was facilitated by knowledge that the DAT was a tool to help PWTB remember to take their medication and improve their adherence [18].

Our study fills an important gap in the literature that assessed the acceptability of the DCA among outpatients. Since the studies from KwaZulu-Natal [10], Uganda [11] and India [16] did not assess DCA, this is a novel finding to our study. Another strength is that our study had a large sample size of 61 interviews and was carried out in three geographically and culturally diverse provinces of South Africa which makes the generalizability high for South Africa. Additionally, the PWTB were interviewed in various months of treatment and were receiving treatment as outpatients which gives us insight into their lived experiences.

Our study did have some limitations. The study evaluated the implementation of the medication monitor within a trial, which, although conducted at routine primary care clinics, was supported by research staff which may have led to an improved experience by the participants. Practical parts of the intervention were that we did not offer the participants any incentives for using the monitors and most home visits were conducted by outreach teams that routinely work in the communities. In the routine setting, home visits are usually conducted by Ward Based Outreach Teams (WBOTs) where the clinic-based TB nurse gives the WBOTs a list of PWTB who have missed their clinic appointments and need to be traced [19]. Given that there is no way of finding out whether the PWTB is still taking their treatment, consideration should be given to integrate aspects of our intervention such as the medication monitor, SMSs and phone calls into the National TB program (NTP) as initial steps to reminding PWTB to take their treatment before WBOTs embark on home visits.

## Conclusion

This study gives us an understanding of the experiences of persons with TB using medication monitors and DCA. Achieving people-centred TB care, including the introduction of DATs and DCA, will require that TB programmes incorporate patient insights to maximize their adoption and effectiveness. Features of the monitor such as size and the alarm which PWTB felt drew attention to their TB status, potentially causing embarrassment and stigma, would need to be addressed before scale-up as they may serve as a barrier to acceptability. It seems the home visits were also not particularly well received and further examination is needed to determine whether these were beneficial. A key highlight from our study is that despite some challenges most PWTB found the medication monitors and DCA as acceptable approaches to supporting medication adherence.
